# Airline Point-of-Care System on Seat Belt for Hybrid Physiological Signal Monitoring

**DOI:** 10.3390/mi13111880

**Published:** 2022-11-01

**Authors:** Xiaoqiang Ji, Zhi Rao, Wei Zhang, Chang Liu, Zimo Wang, Shuo Zhang, Butian Zhang, Menglei Hu, Peyman Servati, Xiao Xiao

**Affiliations:** 1 School of Life Science and Technology, Changchun University of Science and Technology, Changchun 130022, China; 2 Department of Biomedical Engineering, College of Design and Engineering, National University of Singapore, Singapore 117583, Singapore; 3 Department of Materials Science and Engineering, College of Design and Engineering, National University of Singapore, Singapore 117583, Singapore; 4 Department of Imaging, China-Japan Union Hospital of Jilin University, Changchun 130033, China; 5 Department of Electrical and Computer Engineering, University of British Columbia, Vancouver, BC V6T 1Z4, Canada; 6 Department of Electrical and Computer Engineering, College of Design and Engineering, National University of Singapore, Singapore 117583, Singapore

**Keywords:** point-of-care system, wearable biosensors, machine-learning, airline industry security

## Abstract

With a focus on disease prevention and health promotion, a reactive and disease-centric healthcare system is revolutionized to a point-of-care model by the application of wearable devices. The convenience and low cost made it possible for long-term monitoring of health problems in long-distance traveling such as flights. While most of the existing health monitoring systems on aircrafts are limited for pilots, point-of-care systems provide choices for passengers to enjoy healthcare at the same level. Here in this paper, an airline point-of-care system containing hybrid electrocardiogram (ECG), breathing, and motion signals detection is proposed. At the same time, we propose the diagnosis of sleep apnea-hypopnea syndrome (SAHS) on flights as an application of this system to satisfy the inevitable demands for sleeping on long-haul flights. The hardware design includes ECG electrodes, flexible piezoelectric belts, and a control box, which enables the system to detect the original data of ECG, breathing, and motion signals. By processing these data with interval extraction-based feature selection method, the signals would be characterized and then provided for the long short-term memory recurrent neural network (LSTM-RNN) to classify the SAHS. Compared with other machine learning methods, our model shows high accuracy up to 84–85% with the lowest overfit problem, which proves its potential application in other related fields.

## 1. Introduction

Point-of-care systems have received great attention in recent years since it aims to provide diagnosis and therapeutic services with imperceptible devices [1,2,3,4]. Traditional healthcare systems are always lagging and can often only be applied after disease onset or diagnosis [5,6]. Moreover, some chronic diseases require continuous monitoring and long-term nursing, which causes a huge burden on public healthcare resources [7,8,9]. To this end, point-of-care shows its advantages in serving patients with economic and convenient healthcare monitoring, which enable individual diagnosis ahead of time [10,11,12]. Combined with variable wearable devices, such as seatbelts and bracelets [13,14,15], the new-type point-of-care system can be comfortably used by patients in their daily life. As a result, the systems supply sufficient and instant medical services for patients with long-term monitoring, which decrease the medical burden for patients and hospitals, and provide an attractive alternative for customers to enjoy health care in flight. 

For the airline industry, there is nothing more important than the safety of passengers [16,17,18]. However, during air travel, passengers are inevitably exposed to unusual situations such as overweight, weightlessness, low atmospheric pressure, and the fluctuations [19,20]. The occurrence of in-flight medical emergencies is rare while associated costs derived from en route diversion might significantly influence airlines’ budgetary equilibrium [21]. Published data suggest that the incidence of inflight deaths from medical conditions is between 0.3 and 1 per million passengers [22]. For a person who recently underwent chest surgery, it has a 25% expansion of any residual trapped gas with compromise to both circulation and respiration [23]. It is more reasonable for passengers to choose airlines that provide onboard medical services. Considering that associated costs derived from en route diversion might significantly influence airlines’ budgetary equilibrium and the occurrence of inflight medical emergency is rare [21], which has a death incidence between 0.3 and 1 per million passengers [22], the healthcare system can act as an inexpensive way for aviation corporations to reduce the risks of diversion. For some special customers, such as the persons who recently underwent chest surgery recently and therefore have a 25% expansion of any residual trapped gas with compromise to both circulation and respiration [23], it is more reasonable for them to choose airlines that provide onboard medical services. The growing population of older people suffers more medical emergency under these extreme conditions because the elders are often accompanied by various cardiovascular disease [24,25]. Multiple assessment methods based on ECG signals have been applied for the pilot to observe the physiological characteristics during flight operation [26]. Current wearable monitoring equipment on the market for pilots mainly uses belt type and cap type wearable physiological parameters monitoring equipment; belt type equipment can measure and record parameters such as ECG, respiration, and three-phase acceleration in real time, while cap type equipment can measure and record parameters such as head pulse signal and blood oxygen saturation in real time. These devices are usually only given to pilots and captains of dedicated aircrafts. Therefore, retrofitting an existing long-distance transport seat into a novel and modern standardized point-of-care system for general passengers is necessary and offers significant cost savings [21,27].

Sleeping on long trips is almost inevitable, so it makes sense to monitor sleep-related disorders during the flight. Among sleep-related disorders, SAHS is highly prevalent in the general population and is associated with an increased risk of hypertension, diabetes, coronary disease, stroke, and myocardial infarction [28]. However, 75–85% of patients with SAHS are undiagnosed and untreated, which causes irreparable consequences [29,30]. Since the risk of death is associated with SAHS, potential patients are suffering a shortage of being diagnosed. The severity of SAHS is assessed by the apnea-hypopnea index (AHI), defined as the number of apnea and insufficient breathing per hour during sleep [31,32]. In addition, the standard diagnostic test of SAHS is done by polysomnography (PSG) [33]. In clinical settings, patients go to a professional sleep laboratory to complete the acquisition of several signals under the guidance of professional staff, mainly ECG, electroencephalography, electromyography, respiratory airflow signal, blood oxygen saturation, nasal pressure, snoring sounds, etc. Abnormalities of airflow signals or ECG signals in the sensors are used as a basis for clinical judgment. This method quantitatively analyzes the sleep quality of patients and then accurately diagnoses the incidence of SAHS. 

Herein, we suppose a point-of-care system with wearable chest belts for detection of ECG, breath, and motion signals, combined with a designed algorithm to classify SAHS for airlines. This work proposes a system to detect SAHS through a wearable device based on flexible dry ECG electrodes and a machine learning-based classifier algorithm. The signal acquisition terminal is a smart chest band consisting of flexible ECG electrodes and a control box that integrates various signal processing circuits and Bluetooth modules. The wearable ECG electrodes are used to detect ECG signal, while the control box with control circuits is used to detect tri-axis acceleration, and flexible piezoelectric material breathing coil belt is used to detect the breath signal. The detected signals are dealt with by these circuits and then transferred by the Bluetooth module to the Microcontroller Unit (MCU). These signals can then be dealt with by our designed algorithm, which includes the ECG signal denoising, RR interval data extraction, and extraction and selection of HRV features. Then, the characterized signals are analyzed through our designed LSTM to diagnose whether the user is a potential SAHS patient. The results show that the average recognition accuracy of the ECG-SAHS discriminant model is up to 84–85%. Besides that, the chest band design and the material of the electrode made longtime SAHS detection more comfortable, which is especially suitable for transoceanic flights.

Creativity:

1. Originality: Retrofitting existing safety belts, first extending of the point-of-care system on the aircraft to passengers besides pilots.
2. Feasibility: The cost of the equipment is lower than nearly 90% of commercial products since our design is a cost-saving approach by retrofitting of existing safety belts.
3. Accuracy: With the LSTM-RNN based algorithm, the average recognition accuracy of the model is up to 84–85%.
4. Extensibility: Data of hybrid ECG, breathing, and motion signals can also be used for monitoring other diseases.

## 2. Design and Methods

**Figure 1:**
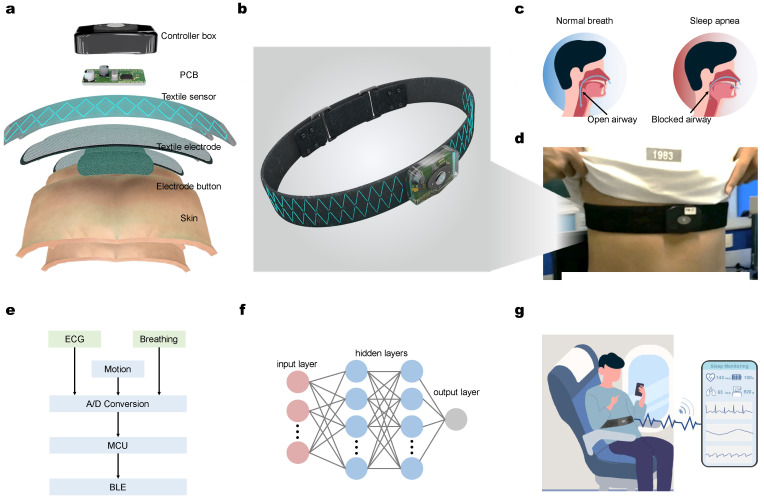
Airline point-of-care system on seat belt. (**a**) Illustration of the layered hardware structure of our designed chest band and (**b**) the fabricated chest band device. (**c**) Aspiration difference between normal breath and sleep apnea caused by airflow. (**d**) Physical photos of the chest belt on a human subject. (**e**) Working flowchart of the monitoring system with hardware and software. (**f**) Neural network that applied in the system as a classifier. The red nodes represent input layer, the blue nodes represent hidden layers, and the gray node represent the output layer in the network. (**g**) Schematic of airline point-of-care monitoring system in the user’s client with Bluetooth-based data transmission to the mobile terminal for hybrid physiological signal detection.

At the hardware design aspect, the system contains a controller box, printed circuit board (PCB), textile electrode, and electrode button on the surface of human skin [34]. The connection of these layer structures is displayed in Figure 1a, while the well-fabricated device and wear model is shown in Figure 1b. Figure 1c indicates that the cause of sleep apnea is the obstruction of throat airflow, which can be further explained as the different aspirations between normal breath. Therefore, we designed the wearing position on the wearer’s chest for better detection of breathing-related signals as Figure 1d shows.

The design of software includes both the ECG signal and the breathing signal. Meanwhile, the motion capture is added into the system as the final classification criterion considering the movement of the user. After A/D conversion, the signal is processed to MCU and then transferred by Bluetooth module to the user’s client on smartphones. The users could access their sleep assessment through the mobile terminal, which is presented in Figure 1e. A learning-based model will classify the user’s aspiration model in Figure 1f. As stated before, the design of our system aims at the application of long-distance traveling. The ECG electrodes are connected to a chest band which could be integrated into safety belts to make the detection comfortable, as Figure 1g shows.

### 2.1. Hardware Design and Signals Acquisition

**Figure 2:**
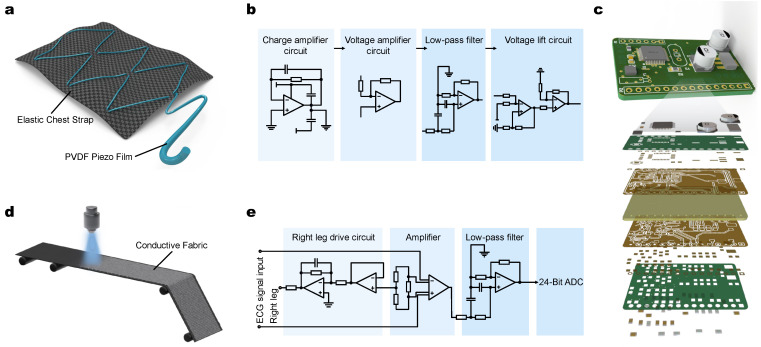
Architecture of the components of airline point-of-care system. (**a**) Fabrication process of breathing coil in elastic chest strap of the wearable terminal, the green line represents PVDF. (**b**) Breathing signal conditioning circuit pattern, including charge amplifier, voltage amplifier, low-pass filter, and voltage lift circuit. (**c**) PCB board displayed in layers. (**d**) Fabrication process of flexible ECG electrodes: from the inner side are the basal layer, the elastic support layer and the conductive fabric, respectively. (**e**) ECG signal conditioning module including right leg drive circuit, amplifier, and low-pass filter, finally 24 Bit ADC.

The flexible dry ECG electrodes with good biocompatibility are selected for the wearable terminal, which is also soft, washable, comfortable, durable, reusable, and easy to integrate with clothing [35,36,37]. Meanwhile, the sensor close to the skin could collect complete and reliable signals. The wearable terminal consists of single lead and double electrodes, which aim to simulate the standard 1-lead to realize the acquisition of ECG signal. As Figure 2a shows, the elastic chest strap is used to fix the flexible electrode to human skin. The fabrication embeds polymers such as polyvinylidene difluoride (PVDF) and other material to form piezoelectric film into the elastic chest strap of the wearable terminal to form a breathing coil, and the front-end collection of human breathing signals is realized through the breathing coil [38,39,40]. The piezoelectric film is subjected to force by human respiration and periodic respiratory rhythm deformation [41]. The surficial charge generated by the piezoelectric film also changes. Human respiration stresses the piezoelectric film and produces periodic deformation with the respiratory rhythm, and the amount of charge generated by the piezoelectric film also changes. By extracting the surface charge of the piezoelectric film and converting it into voltage amount, and then after signal conditioning, the respiratory signal can be obtained. Therefore, the acquisition method used in this paper is the piezoelectric breath tracing method. 

Compared with the patch electrode, the flexible electrode can fit closely with the skin surface and change its shape to maintain continuous contact with the skin, which is useful for wearable ECG signal monitoring equipment [42]. The electrodes commonly used in clinical practice are traditional patch electrodes, which can cause skin irritation to the user over a long period of time. In addition, the impedance between the electrode interface and the skin will become larger as the detection time increases, resulting in a decrease in signal sensitivity and signal-to-noise ratio (SNR). Applying flexible fabric electrodes is to attach conductive materials to the fabric through various coating processes and braiding the lead wire on the clothing to prevent the lead wire from winding [43]. The inner side of the elastic chest strap facing the skin is the base layer and the conductive sensing layer. The base layer is made of polyester fiber. The base-layer fabric is wear-resistant, elastic, and easily washable. The conductive sensing layer is made of copper-plated conductive fabric and then blended with acrylic fiber. As a thinner electrode results in a larger area and better ECG signal quality [44], the experimental results show that the flexible electrode has a higher signal-to-noise ratio and higher stability than the conventional electrode [45]. Figure 2d shows the flexible ECG electrodes. From the inner side of the elastic chest strap facing the skin in the direction of the base layer, the elastic support layer, and the conductive fabric sensing layer, in turn, greatly reduces the thickness of the electrodes and improves the wearing comfort and is suitable for dynamic and long-time ECG monitoring. The system sews the two flexible fabric electrodes closely to the left and right inner side of the elastic chest strap near the controller box so that they can fit close to the skin, and the ECG signals collected using the flexible fabric electrodes need to be processed before the next step.

The circuit pattern of the wearable signal acquisition terminal is shown in Figure 2b. The signal collected through the breathing coil is relatively weak, with a frequency of 0.1–1 Hz, easily disturbed by various noises. Therefore, the breathing signal needs to be conditioned, including signal charge amplification, voltage method, low pass filtering, and voltage boosting, and the system designs a conditioning circuit for the breathing signal based on the TLV2464 chip, which is used to extract the effective breathing signal [46]. The charge amplification circuit of the respiratory signal and the voltage amplification circuit are designed through the first op-amp and the second op-amp of the TLV2464 chip, respectively. The amplification of the amplifier circuit is 80 times. A second-order active low-pass filter circuit is designed to filter out high-frequency noise with a cutoff frequency of 88.46 Hz. A voltage boost circuit is designed based on the fourth op-amp of the TLV2464 chip to boost the signal to a standard that the microprocessor can recognize, and the signal is transmitted to the microprocessor through the TLV_OUT pin. Use the A/D module in the microprocessor for analog-to-digital conversion processing.

The signal acquisition terminal consists of a controller box, a biomedical sensor, and an elastic chest strap, which enables 8 h of continuously monitoring. The extended monitoring time of the flexible electrode shows almost no influence on the signal sensitivity and the SNR. The collected ECG signal is processed by the circuit and sent to the microprocessor, and then transmitted to the computer through a Bluetooth module for further analysis and processing. The removable controller box contains a power supply, a communication module, a signal processing module, the main control chip, and the memory, which is integrated on a fast-prototyping PCB board that patterned using a UV laser system (LPKF; Protolaser U4), as shown in Figure 2c. Four electrode buttons are mounted on the elastic chest strap. The biomedical sensor is a flexible ECG electrode stitched tightly to the elastic chest strap to ensure continuous contact with the skin surface. The control box of the belt comes with a three-axis acceleration sensor MPU9150 to collect motion signals, MPU9150 sensor role is to measure the *X*, *Y*, *Z*-axis acceleration signal into electrical signals, through the analog-to-digital conversion and then convert the measured analog signal into a digital signal, so as to complete the extraction of human body position information of the subject.

In collecting ECG signals, certain noise will be introduced, requiring the circuit to have a high common-mode rejection ratio (CMRR) and high-precision analog-to-digital conversion (ADC). Therefore, the high-precision medical chip ADS1292 is used to design the ECG signal conditioning circuit, which has a built-in amplifier circuit and a high-resolution ADC and can reduce common mode interference [47]. The pin configuration and the peripheral circuit of ADS1292 are shown in Figure 2e. The pin configuration and peripheral circuit diagram of ADS1292 can be found in the supplementary Figure S1. After the ECG signal is input through the CH2 channel, the ADS1292 will amplify the ECG signal and perform A/D conversion through the internal modulation circuit, where the gain is set to 1000. The system directly feeds back the output of the right leg drive to the two detecting electrodes to realize the function of the right leg drive circuit. The SPI communication interface transmits the ECG signals after ADC to the microprocessor (EFM32), in which the ADS1292 is the slave device for communication. EFM32 is responsible for the overall control of the system, including initializing, configuring Bluetooth, ADC, and reading and storing ECG data. The photo of the industrial produced PCB is shown in Figure S2.

After we built the signal acquisition terminal, 18 adult volunteers aged between 20 and 39, including 9 males and 9 females, were selected for experimental verification. They had good sleep quality and did not suffer from respiratory and heart diseases. The subjects knew the content of the experiment in advance and agreed with it. The subjects were required to wear the terminal for a 3 h sleep experiment in a quiet room with both normal breathing and apnea. During the experiment, each subject was asked to discontinue breathing randomly, and the time and duration of artificial apnea were recorded. The details of the 18 adult volunteers can be found in the Supplementary Material Table S1.

The ECG signal collected by the terminal was transmitted to the computer by a Bluetooth module. The collected data then dealt with denoising and classification in the next steps. The details of collection methods will be discussed in the next section.

We computationally extracted three shallow feature signals of ECG: the RR interval signal (RRI), the R peak amplitude sequence signal (RAMP), and the ECG-derived respiratory (EDR), and used them as features for the detection of SAHS. It is important to note that the EDR signal is obtained starting from the R peaks detected by the Pan–Tompkins algorithm. It is just a pseudo-breathing signal. The calculated EDR signal has a certain amplitude error compared with the real respiratory signal, which cannot completely replace the respiratory signal. However, the EDR signal is an effective feature for detecting SAHS. Although SAHS have many subcase categories due to causes, since they have similar performance, we did not distinguish these subcases here.

These three signals are calculated using only the R-peak position index (RP_idx) and the denoised ECG (ECGF, F is just a marker to distinguish from denoised ECG and ECG, no special meaning) signal. The RRI signal refers to the time interval between adjacent R-peak positions. The RRI signal refers to the time interval between adjacent R-peak positions as Equation (1) shows.

(1) $$RRI=RP\_idx_{i+1}-RP\_idx_{i}$$

The RAMP signal refers to the amplitude sequence of the R-peak position in the denoised ECG signal ECGF in Equation (2), where *L* is the length of the current R-peak position index.

(2) $$RAMP_{i}=ECGF\left[RP\_idx_{i}\right],0<=i<=L$$

The EDR signal can show the current respiratory airflow and is often used in the detection of SAHS. In this paper, the kurtosis of the ECG signal between the adjacent R peaks and the ECG signal between the adjacent R peaks is calculated to obtain the sampling of the EDR signal at the current R peak, and then the complete EDR signal is obtained by sample bar interpolation. First, the kurtosis of the ECG signal between the adjacent R peaks is calculated as Equation (3) [48]

(3) $$K_{i}=\frac{\left(1/n\right)\sum_{t=0}^{J_{i}-1}{\left(ECGFS_{it}-\bar{ECGFS_{it}}\right)}^{4}}{\left(1/n\right)\sum_{t=0}^{J_{i}-1}{\{{\left(ECGFS_{it}-\bar{ECGFS_{i}}\right)}^{2}\}}^{2}}-3,\;0<=i<=L-1$$

(4) $$J_{i}=RP\_idx_{i+1}-RP\_idx_{i}+1$$

where $ECGFS_{i}$ is the segment of the signal ECGF from $RP\_idx_{i}$ to $RP\_idx_{i+1}$, $\bar{ECGFS_{i}}$ is the arithmetic mean of $ECGFS_{i}$, and $J_{i}$ is the length of the $ECGFS_{i}$ signal segment, $J_{i}$ can be calculated as Equation (4). After obtaining the kurtosis of the ECG signal between adjacent R peaks, the peak EDR signal sampled on that segment of the ECG signal can be obtained as Equation (5),

(5) $$EDR_{i}={\{\frac{{\left(\sigma^{4}+\left(\gamma^{4}-3\sigma^{4}\right)K_{i}\right)}^{1/2}-\sigma^{2}}{\gamma^{4}-3\sigma^{4}}\}}^{1/2},\;0<=i<=L-1$$

(6) $$\gamma^{4}=\exp\{\left(ECGFS_{i}-\exp(ECGFS_{i}\right){)}^{4}\}$$

where *σ* is the standard deviation of the signal $ECGFS_{i}$, $\gamma^{4}$ is the fourth-order central moment of the signal $ECGFS_{i}$ that can be calculated as Equation (6), exp is the expectation, and K is the kurtosis of the signal $ECGFS_{i}$. At last, the EDR signal can be obtained by using spline interpolation for the discrete EDR signal.

### 2.2. ECG and Breathing Signal Collecting and Processing

**Figure 3:**
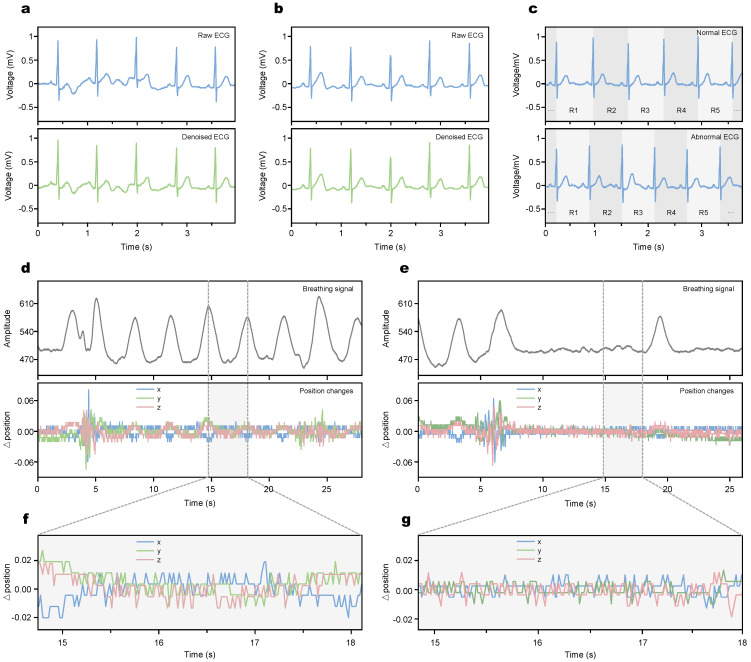
The display of hybrid physiological signal. (**a**) ECG signal before and after denoising at normal breath model. (**b**) ECG signal before and after denoising without apnea occurs. (**c**) ECG signals in normal and apneic RR classification. (**d**) Breathing signal and position signal in normal condition. (**e**) Breathing signal and position signal when apnea occurs. (**f**,**g**) Details of local fragments of position signals.

The ECG signals were collected at a normal sleep state by asking the subjects to close their eyes and breathe normally. The data were filtered by cutting the beginning and denoising to avoid subject psychological factors or other interference, finally the comparison of raw and denoising data under the normal sleep model can be observed as a waveform in Figure 3a. Although there are some small disturbances caused by body movement and electrode sliding, such as baseline drifts and interference, the ECG characteristics can still be clearly identified through the waveform in raw data. Through the noise reduction process, the ECG characteristics of the signal become more obvious and easier for the subsequent classification process. Then, the subjects were asked to simulate sleep apnea by changing the breath model. After a period of normal breathing, when the subjects’ breathing was stable, their breath situations were measured and then randomly held their breath for 10 s or more. When the airflow through the mouth and nose stopped and the chest and abdomen breathing disappeared, the apnea could be confirmed. The ECG signal waveforms of the subjects under sleep apnea are shown in Figure 3b, which also contain the comparison between raw ECG data and denoising ECG. Then, the denoised ECG signals of normal and abnormal waveforms were dealt with time between the successive R peaks to obtain the R-R interval in Figure 3c. Then, the HRV time-domain and frequency-domain analyses from RRI data were processed, which could extract and select features for further classification. 

For the breathing signal, the breathing and position changes in a three-dimensional direction were collected and analyzed. The breathing data could help analyze the breathing signal to verify the situation of the user because when SAHS occurs, the breathing and breathing signal will also vary to a lower stable waveform [49]. Our system is designed to detect the breathing and position signals on two scales. For the large-scale time, in Figure 3d,e, the waveforms show the difference between normal conditions and when SAHS occurs. In addition, the R-peak in the ECG signal can characterize the respiratory activity, with inspiration and expiration corresponding to a decrease and an increase in the R-wave peak, respectively. During the onset of OSA, the corresponding R-wave peak will be lower than normal due to the lungs inhaling harder than normal. The R-peak interval of the ECG signal in the normal segment is mainly between 0.8 and 1.0 s, and the R-peak amplitude fluctuates in the range of 0.6 and 0.8 mV. In contrast, the R-peak interval of the ECG signal in the OSA fragment was mainly between 1.1 and 1.2 s, which was significantly larger than the R-peak interval of the normal ECG signal. In addition, the R-peak amplitude fluctuated slightly around 0.6 mV, which was significantly lower than that of the normal ECG signal. Generally, when the abnormal waveforms last for 8 s, it means that the SAHS occurs. The large-scale waveform that directly reflects the user’s situation suffers obstacles from the abnormal stable waveform’s appearance in Figure 3e, while the system also designed a small-scale detection for accurate analysis. In Figure 3f,g, the change of position is recorded in terms of three-dimensional displacement, which has an accuracy of less than 0.01. Our design of hardware of flexible electrodes enables detection accuracy. This accuracy ensures the waveforms on a large scale with credibility and operability. Although these two kinds of signals contribute a little to the system for the function of verification of SAHS, they will be helpful for other diseases’ point-of-care detection.

### 2.3. Machine Learning-Enabled SAHS Recognition

To distinguish the ECG signal, the classification algorithm can be used to deal with the data efficiently. The above signals are not unstable for a typical man, and by coupling with modern computer science technology [49], multiple methods including Support vector machine (SVM), Random Forest Classifier (RFC) [50], Decision Trees (DT), K-Nearest Neighbour (KNN) [51], Adaptive Boosting Algorithm (ADABoosta), Gaussian naïve Bayes Classifier (GNB), Quadratic Discriminant Analysis (QDA), and BP are used to classify these data. Statistical methods mainly use mathematical and statistical analysis methods to establish models such as autoregressive models, binary tree classification models, and classify and analyze signals through them. However, due to the characteristics of ECG signals, it is difficult to establish a reasonable classification model and ensure the accuracy of it. The deep convolutional neural network (CNN) model seems to be a feasible choice as the sample data in this paper are small, but the severe overfitting problem of it is yet to be solved. 

**Figure 4:**
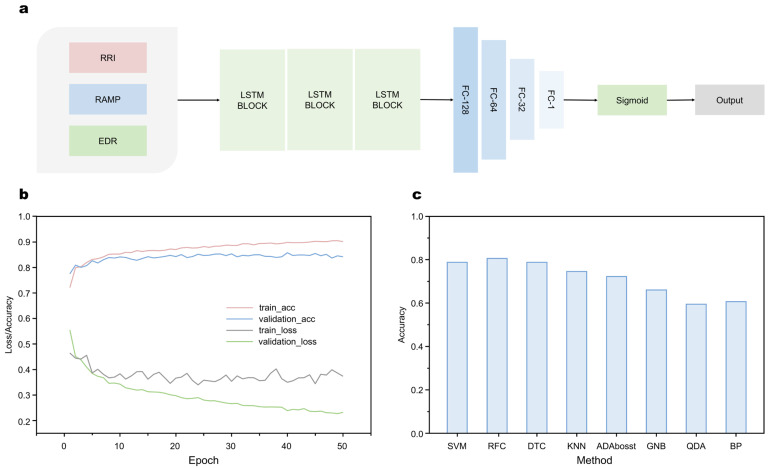
Structure and performance of SAHS detection system. (**a**) Structure of LSTM-RNN. (**b**) Loss/accuracy versus epoch for train and validation sets. (**c**) Comparison of different methods in terms of accuracy.

Here we choose (LSTM-RNN) to avoid the overfitting problem. LSTM has the function of memory effect, which is more efficient for dealing with time series data. As an extension model of RNN, it also solves the Vanishing Gradient problem of gradient back-propagation process due to gradual reduction. The working mechanism of LSTM-RNN is presented in Figure 4a. Three manual features were first extracted from the ECG signal as RR interval signal RRI, R peak amplitude sequence signal RAMP, and respiratory signal EDR derived from the ECG signal (not the respiratory signal acquired directly by the instrument), then fed into the model. Because traditional methods only use the RRI signal as the input signal of the neural network, only the variation of heart rate can be reflected, and the variation of respiratory airflow and the variation of chest and abdomen during breathing. The model consists of three LSTM loop layers and four fully connected layers with nodes 128, 64, 32, and 1. The output is either 0 or 1 to determine the presence or absence of apnea in the one-minute ECG signal segment. The training results of 50 epochs were recorded in supplementary material Table S2, and these data are presented in the form of a graph in Figure 4b. Despite suffering from overfitting starting at around 10 epochs, the model achieves an accuracy of over 80 percent in Apnea-ECG database [52]. The detail of Apnea-ECG database could be found in the SI Apnea-ECG database section.

Binary cross-entropy as loss function was given as follows,

(7) $$L\left(Y,P\right)=-\sum_{x}Y_{x}\mathrm{lg}(P_{x})$$

where $P_{x}$ represents the prediction of the LSTM-RNN network for ECG fragment x, i.e., the Y of the output in the LSTM-RNN network, represents the true label of ECG fragment x. The final trained loss and accuracy are approaching 0.4 and 0.9, whereas the validation loss and accuracy are approaching to 0.8 and 0.2. Compared with other networks stated before, SVM, RFC, DTC, KNN, ADAboosta, GNB, QDA, and BP, our model gets the highest accuracy, which is shown in Figure 4c. The details of comparison of different methods in terms of accuracy could be found in supplementary material Table S3. For a fair comparison, the same manual features and training strategies were used.

To evaluate the classifier, Se, Sp, +PV, Acc, and F-score were applied as judgment categories, where Se represents the correct recognition rate of SAHS, Sp represents the detection rate of normal samples, +PV represents the actual response rate determined as SAHS, Acc represents the detection accuracy of any sample, and F-score is a comprehensive evaluation of the classification performance of our model. The detail of each evaluation criteria could be found in the supplementary material Table S4. Finally, we use the model of this paper to test its performance on the collected 18 subjects. The result shows high accuracy up to 84–85%.

## 3. Conclusions

In this paper, we build a point-of-care system model based on ECG electrode, flexible piezoelectric materials, and a control box to achieve ECG, breathing, and position signal detection. The diagnosis of SAHS for flight traveling is taken as a long-term application of this paper. Results show that the cost of the equipment is lower than nearly 90% of commercial products, while the average recognition accuracy of the model is up to 84–85%. The system originally retrofits existing safety belts to our system, firstly expanding point-of-care system from pilots to regular passengers in the post-epidemic era. By using flexible materials in the hardware design, the device is highly fitted to the human body, thus guaranteeing the accuracy of the breathing signal obtained by the piezoelectric sensor. At the same time, the data features extracted from the mixed acquisition of ECG, breath, and motion signals can reduce the diagnosis error rate since these signals reflect the variations of airflow and chest. At last, LSTM-RNN is applied to avoid the overfitting problem while ensuring high classification accuracy. The designed system also has important research significance and broad application prospects since the ECG, breathing, and motion signals could be applied to diagnose other diseases. It is worth believing that the system could have potential applications in future human aeronautics and astronautical space exploration.

## Data Availability

All data generated or analyzed during this study are included in this published article and its supplementary information files.

## Supplementary Materials

The following supporting information can be downloaded at https://www.mdpi.com/article/10.3390/mi13111880/s1, Table S1: The Screening Model Test Results; Table S2: Loss/accuracy versus epoch for train and validation sets; Table S3: Comparison of different methods in terms of accuracy; Table S4: Classifier evaluations index; Table S5: Cost of each component for the point-of-care system in detail.; Figure S1: Pin configuration and peripheral circuit diagram of ADS1292; Figure S2: Photo of fabricated PCB.
