# Supplementary material for: Airline Point-of-Care System on Seat Belt for Hybrid Physiological Signal Monitoring
Source: Micromachines (Basel). 2022 Nov 1;13(11):1880. doi: 10.3390/mi13111880 (PMC9694689; doi:10.3390/mi13111880)
Supplement: Supplementary file 1 [file micromachines-13-01880-s001.zip › micromachines-1997246-supplementary.pdf]

Supplementary Information: Airline Point-of-Care System on Seat Belt for Hybrid Physiological Signal Monitoring

Xiaoqiang Ji [1,†], Zhi Rao [1,†], Wei Zhang [2,†], Chang Liu [3], Zimo Wang [3], Shuo Zhang [1], Butian Zhang [4], Menglei Hu [5,*], Peyman Servati [5], Xiao Xiao [6,*]

[1] School of Life Science and Technology, Changchun University of Science and Technology, Changchun 130022, China
[2] Department of Biomedical Engineering, College of Design and Engineering, National University of Singapore, Singapore 117583, Singapore
[3] Department of Materials Science and Engineering, College of Design and Engineering, National University of Singapore, Singapore 117583, Singapore
[4] China Japan Union Hospital of Jilin University, Changchun 130033, China
[5] Department of Electrical and Computer Engineering, University of British Columbia, Vancouver, BC V6T 1Z4, Canada
[6] Department of Electrical and Computer Engineering, College of Design and Engineering, National University of Singapore, Singapore 117583, Singapore
* Correspondence: mengleih@ece.ubc.ca; xiao.xiao@u.nus.edu.
† These authors contributed equally to this work.

Table S1. The Screening Model Test Results

Number of subject	Simulated normal state (minutes)	Simulated apnea (minutes)	Correctly detected by the model at the normal state (minutes)	Correctly detected by the model at the apnea state (minutes)
Male 1	140	40	121	34
Male 2	134	46	112	34
Male 3	128	52	108	46
Male 4	117	63	98	52
Male 5	124	56	102	41
Male 6	141	39	118	32
Male 7	114	66	105	43
Male 8	130	50	114	38
Male 9	140	40	118	33
Female 1	145	35	123	25
Female 2	135	45	113	38
Female 3	132	48	118	41
Female 4	143	37	119	30
Female 5	123	57	106	46
Female 6	120	60	100	47
Female 7	133	47	113	41
Female 8	125	55	98	48
Female 9	127	53	112	41
Average	130.61	49.39	111	39.44

Table S2. Loss/accuracy versus epoch for train and validation sets.

epoch	train_acc	train_loss	validation_acc	validation_loss
1	0.720068	0.5551379	0.7746267	0.464238003
2	0.79807	0.4504302	0.808508	0.443691787
3	0.802456	0.4377426	0.79966116	0.440567849
4	0.81862	0.4096381	0.8068139	0.454980261
5	0.830524	0.3846521	0.82595056	0.386880132
6	0.830524	0.3734832	0.8171665	0.401154585
7	0.840549	0.3684623	0.82971513	0.381569703
8	0.850949	0.3462507	0.8383737	0.367486143
9	0.851638	0.347045	0.83655417	0.370268121

Apnea-ECG Database

Description

The data consist of 70 records, divided into a learning set of 35 records (a01 through a20, b01 through b05, and c01 through c10), and a test set of 35 records (x01 through x35), all of which may be downloaded from this page. Recordings vary in length from slightly less than 7 hours to nearly 10 hours each. Each recording includes a continuous digitized ECG signal, a set of apnea annotations (derived by human experts on the basis of simultaneously recorded respiration and related signals), and a set of machine-generated QRS annotations (in which all beats regardless of type have been labeled normal). In addition, eight recordings (a01 through a04, b01, and c01 through c03) are accompanied by four additional signals (Resp C and Resp A, chest and abdominal respiratory effort signals obtained using inductance plethysmography; Resp N, oronasal airflow measured using nasal thermistors; and SpO2, oxygen saturation). More information about Apnea-ECG databas： https://physionet.org/content/challenge-2000/1.0.0/

Label

One annotation per minute is used to indicate the presence or absence of apnea in that minute. One minute was used as the criterion for segmentation, i.e., one hour was segmented into 60 segments, and the labelling was based on whether apnea was present in each segment: if apnea was present in a minute segment (no matter how many times), the labelling was A; if no apnea was present, the labelling was N.

Note: These reference notes were made by human experts on the basis of a combination of Resp C for chest breathing, Resp A to indicate abdominal breathing, Resp N for oral and nasal airflow, and SpO2 for blood oxygen saturation.

Table S3. Comparison of different methods in terms of accuracy

Methods	Accuracy
SVM	0.78747
RFC	0.80586
DTC	0.78747
KNN	0.7454
ADAbosst	0.72172
GNB	0.66064
QDA	0.59488
BP	0.6061

Table S4. Classifier evaluations index

Parameters	Equations
Se	$Se = \frac{TP}{TP + FN}$
Sp	$SP = \frac{TN}{TN + FP}$
+PV	$+PV = \frac{TP}{TP + FP}$
Acc	$Acc = \frac{TP + TN}{TP + Fn + TN + FP}$
F-score	$F = \frac{2 \times Se \times Sp}{Se + Sp}$

Table S5. Cost of each component for the point-of-care system in detail.

Item	Belt	PCB	Sensors	Fabrication	Total
Cost ($)	2.2	2.63	150.62	6.01	161.46

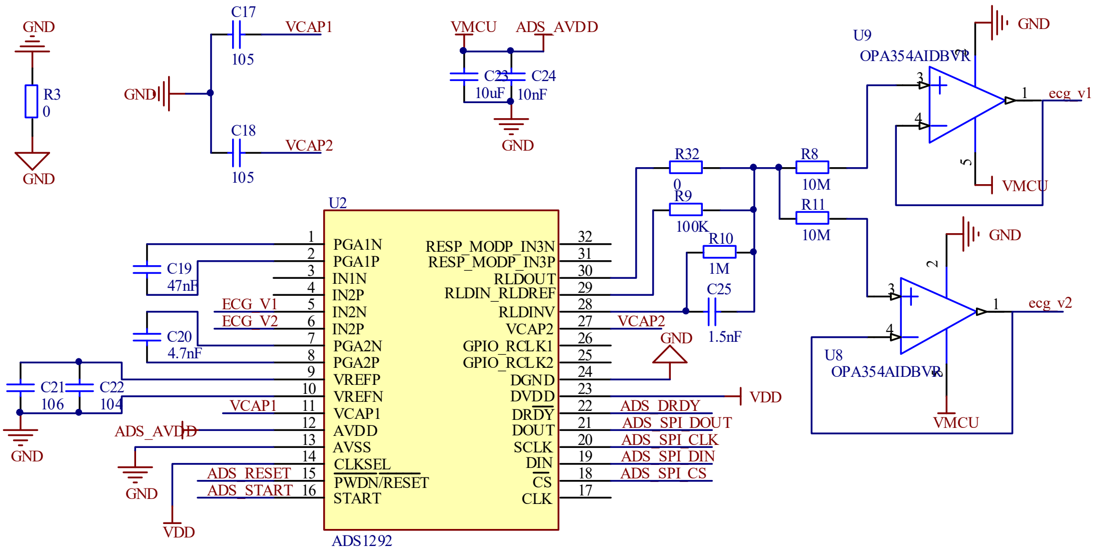

Figure S1 Pin configuration and peripheral circuit diagram of ADS1292

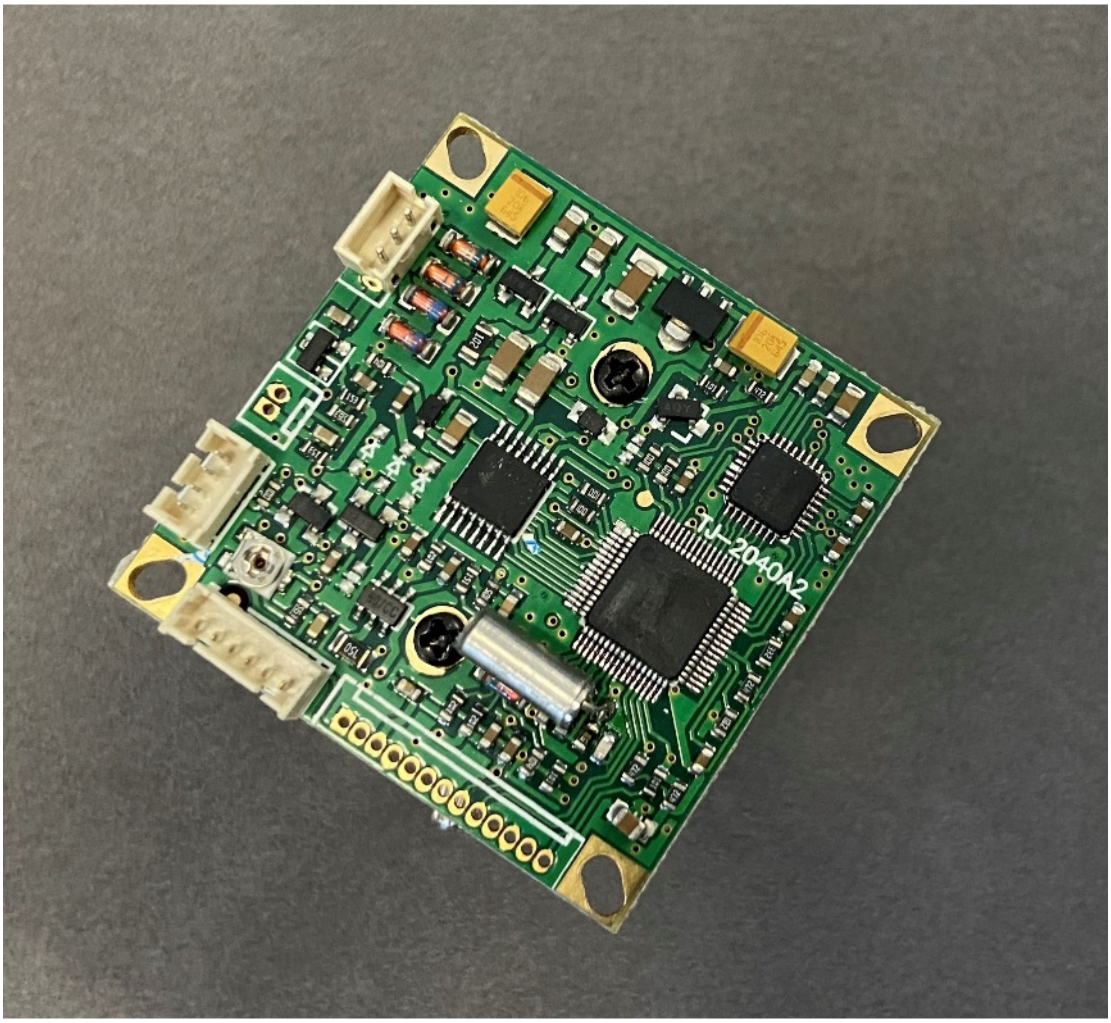

Figure S2 Photo of fabricated PCB
